# Assessment of color stability and translucency of various CAD/CAM ceramics of different compositions and Thicknesses: An in vitro study

**DOI:** 10.1016/j.sdentj.2024.05.001

**Published:** 2024-05-03

**Authors:** Passent Ellakany, Nourhan M. Aly, Shahad T. Alameer, Turki Alshehri, Shaimaa M. Fouda

**Affiliations:** a Department of Substitutive Dental Sciences, College of Dentistry, Imam Abdulrahman Bin Faisal University, Dammam, Saudi Arabia; b Department of Pediatric Dentistry and Dental Public Health, Faculty of Dentistry, Alexandria University, Alexandria, Egypt; c College of Dentistry, Imam Abdulrahman Bin Faisal University, Dammam, Saudi Arabia

**Keywords:** CAD/CAM, Dental Ceramics, Color, Thickness, Staining Solution

## Abstract

**Background:**

Translucency and optical characteristics significantly affect the aesthetics of ceramic restorations. The introduction of new virgilite crystals to novel ceramic advanced lithium disilicate through a matrix firing process enhances the material’s strength and aesthetic quality according to the manufacturer.

**Materials and Methods:**

Four types of CAD/CAM ceramics; lithium disilicate (LD), leucite reinforced (LR), advanced lithium disilicate (ALD), and zirconia-reinforced lithium silicate (ZLS) exposed to different staining solutions (tea, cola, coffee, and distilled water). Each ceramic type had 120 specimens, divided into subgroups based on thickness (0.5 mm, 1 mm, and 1.5 mm). Color changes (ΔE00) and translucency parameters (TP00) were measured after thermocycling, and spectrophotometry was employed to evaluate ΔE00 and TP00 after 1, 7, and 30 days of exposure to the staining solutions.

**Results:**

ZLS exhibited the lowest color stability, while lithium LD and ALD displayed the highest. Thickness significantly affected color stability, with 0.5 mm thickness showing the least change. The duration of exposure also impacted color changes, with 30 days causing the most significant shift. LD demonstrated the highest translucency. Coffee and tea induced more pronounced reductions in translucency and color stability compared to the control group.

**Conclusions:**

Material structure, thickness, staining solution, and exposure duration significantly influenced the color stability and translucency of CAD/CAM ceramics, with LD and ALD demonstrating superior color stability and LD exhibiting the highest translucency.

## Highlights

• Lithium disilicate and advanced lithium disilicate CAD/CAM ceramics displayed the highest translucency and color stability.
• Thickness significantly affected color stability, with 0.5 mm thickness showing the least color change unlike 1.5 mm thickness.
• The duration of exposure to staining solutions also impacted color changes, with 30 days causing the most significant shift.
• Coffee and tea induced more pronounced reductions in translucency and color stability among all tested CAD/CAM ceramics.
• Novel advanced lithium disilicate CAD/CAM ceramics show that it can be used to fabricate esthetic restorations as dental veneers, all ceramic restorations, and endocrown of optimum shade matching and color stability.

## 1 Introduction

CAD/CAM ceramics may potentially exhibit color variations in response to endogenous or exogenous factors. Endogenous influences encompass the hardness and polishability of the material, material aging, polymerization time, and resin matrix composition. Exogenous factors include dietary choices and smoking habits. Understanding the optical properties of CAD/CAM ceramics is crucial for anticipating their long-term clinical performance (Quek et al., 2018, Lauvahutanon et al., 2016, Aydın et al., 2020).

LD and ZLS are two types of glass-ceramics. Owing to the superior mechanical and aesthetic traits, LD ceramic is often considered the preferred material for posterior and anterior restorations (Ellakany et al., 2021a, 2023a, Freitas et al., 2023). In contrast, ZLS inclusion of zirconia particles and silicate crystals within the glassy matrix allowed their usagein implant-supported restorations as well as aesthetic tooth restorations, such as veneers (Ellakany et al., 2023a, Ellakany et al., 2023b, Zarone et al., 2021).

LR ceramics exhibit outstanding mechanical strength, and superior optical properties make them suitable for use in veneers and anterior crowns (Ellakany et al., 2021a, 2023a, Kose et al., 2023). ALD was recently introduced to the dental industry. This material comprises a unique crystal particle known as virgilite, which is incorporated into the lithium disilicate crystals to form lithium aluminum silicate ceramics (Demirel et al., 2023, Rosentritt et al., 2022, Gunal and Ulusoy, 2018). According to the manufacturer’s assertions, the introduction of new virgilite crystals through a matrix firing process enhances the material’s strength and aesthetic quality (Demirel et al., 2023).

There is a notably scarcity of research examining the potential variations in the translucency and color of CAD/CAM ALD materials when exposed to various beverages commonly found in the oral environment. Consequently, this study aimed to evaluate the color stability and translucency of various CAD/CAM ceramic materials following exposure to different staining solutions. The null hypothesis of this study is that the composition and thickness of the ceramics do not significantly affect their color and translucency when immersed in various staining beverages.

## 2 Material and Methods

### 2.1 Sample grouping

**Fig. 1:**
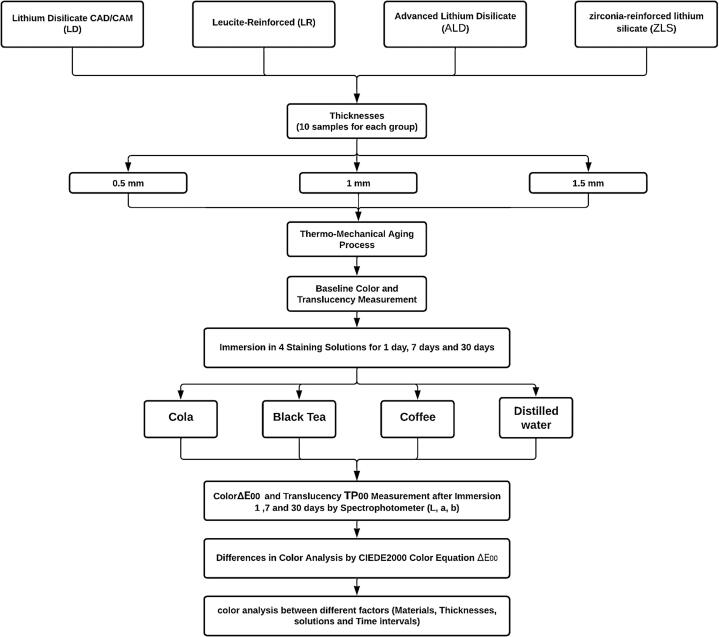
Flow chart of study design.

In this study, four ceramic groups were used: LD (IPS Emax CAD, Ivoclar Vivadent, Schaan, Liechtenstein), LR (IPS Empress CAD, Ivoclar Vivadent, Schaan, Liechtenstein), ALD (Cerec Tessera, Sirona Dentsply, Milford, DE, USA), and ZLS (Celtra Duo, Sirona Dentsply, Milford, DE, USA). All CAD/CAM blocks were of shade A1 low translucency as in Fig. 1.

The sample size was determined with 80 % study power and a 5 % alpha error. In a study by Demirel et al. (2023), the mean color variation (±standard deviation) for LDS, ZLS, and ALD was found to be 27.89 ± 0.81, 20.32 ± 0.89, and 24.79 ± 0.95, respectively. A two-tailed test comparing these means indicated a minimum required sample size of 9 specimens per group, which was increased to 10 to account for potential laboratory processing errors. Thus, the total sample size required was calculated as follows: 10 specimens per group × 4 groups × 3 subgroups × 4 staining solutions, totaling 480 specimens (Charan et al., 2013).

### 2.2 Specimens’ preparation

All CAD/CAM blocks were sliced into three dimensions: 0.5, 1, and 1.5 mm thicknesses, while maintaining their original block dimensions. Sectioning was carried out using a precision cutting machine (Isomet 5000, Buehler, Lake Bluff, IL, USA), with an ample supply of cooling water, in accordance with the International Organization of Standards (ISO standards 6872:2015). Subsequently, the specimens underwent polishing and crystallization as mentioned in previous studies (Ellakany et al., 2021a, Ellakany et al., 2023a, Ellakany et al., 2023b) in the research lab of College of Dentistry, Imam Abdulrahman Bin Faisal University, Dammam, Saudi Arabia.

### 2.3 Thermocycling procedure

The specimens underwent 5000 thermal cycles in a thermocycling machine (Thermocycler THE-1100—SD Mechatronik GmbH, Feldkirchen-Westerham, Germany; Fig. 1). These cycles included temperature fluctuations between 5 °C and 55 °C, with a 30-second immersion in a water bath at each temperature and a 10-second transition between the temperature baths, effectively replicating the effect of six months of aging (Ozen et al., 2020).

### 2.4 Assessment of ceramic translucency parameter (TP)

The initial color readings of all samples were assessed in the CIELAB color space against a white and black background using a spectrophotometer (Color-Eye 7000A Spectrophotometer X-Rite, USA; Fig. 1). The spectrophotometer was configured to operate in the basic shade measurement mode. To ensure accuracy, the spectrophotometer was calibrated at intervals specified by the manufacturer. To minimize potential color value discrepancies, each specimen underwent three repeated measurements, and the results were averaged (Kanat-Ertürk et al., 2020).

$${{TP}_{00}=\left[{\left(\frac{{L\prime }_{B}-{L\prime }_{W}}{K_{L}S_{L}}\right)}^{2}+{\left(\frac{{C\prime }_{B}-{C\prime }_{W}}{K_{C}S_{C}}\right)}^{2}+{\left(\frac{{H\prime }_{B}-{H\prime }_{W}}{K_{H}S_{H}}\right)}^{2}+R_{T}\left(\frac{{C\prime }_{B}-{C\prime }_{W}}{K_{C}S_{C}}\right)\left(\frac{{H\prime }_{B}-{H\prime }_{W}}{K_{H}S_{H}}\right)\right]}^{1/2}$$

“B” and “W” represent the lightness (L′), chroma (C′), and hue (H′) values of the specimens against black and white contrasting sheets, respectively. The rotation function, RT, is applied to address the interplay between C’ and H’ variations in the blue zone. Weighting roles, SL, SC, SH, modify the overall color variation to accommodate shifts in the color variation specimen's position over the B and W backgrounds in L′, a′, b′ coordinates. Additionally, the critical elements, KL, KC, KH, serve as adjustment components for conducting research (Salas et al., 2018).

The translucency parameter was repeated for all the samples after placement in different staining solutions after 1 day, 7 and 30 days. Then, the difference in the translucency was assessed in comparison to the initial measurement before immersion using the same equation.

### 2.5 Color stability test

The tea solution was created using a 2 g tea bag (Lipton Yellow Label) soaked in 200 ml of boiling water. Similarly, the coffee solution was prepared as per the manufacturer's recommendations, using 3.6 g of coffee grounds (Nescafe Classic; Nestle) and 300 ml of boiling water. After a 10-minute steeping period, the solution was extracted through a filter sheet. The Cola solution (The Coca-Cola Company) was made by using a 330 ml can of Cola, and it was gently stirred every 8 ± 1 h. For the control group, distilled water was used (Ellakany et al., 2023c). All the specimens were placed in tightly secured glass containers, each containing 100 ml of solution, at a constant temperature of 37 °C for durations of 1 day, 7 days, and 30 days (Kanat-Ertürk et al., 2020). Subsequently, the specimens were washed with distilled water for 5 min and dried with tissue paper (Alsilani et al., 2022, Ellakany et al., 2023c).

The color characteristics of all the samples were evaluated at 1 day, 7 and 30 days intervals using the spectrophotometer (Color-Eye 7000A Spectrophotometer X-Rite, USA; Fig. 1). The assessment procedure mirrored that of the initial measurements. Each specimen was subjected to three separate measurements and the resulting values were averaged (Ellakany et al., 2023c).

The color deviation (ΔE_00_) between the initial measurements and those taken after 1 day, 7 days, and 30 days of storage were computed for each sample using the following formula:

$$\Delta E_{00}=\sqrt{{\left(\frac{\Delta L*}{K_{L}S_{L}}\right)}^{2}+{\left(\frac{\Delta C*}{K_{C}S_{C}}\right)}^{2}+{\left(\frac{\Delta H*}{K_{H}S_{H}}\right)}^{2}+R_{T}\left(\frac{\Delta C*}{K_{C}S_{C}}\right)\left(\frac{\Delta H*}{K_{H}S_{H}}\right)}$$

The components K_L_, K_C_, K_H_ are the tested elements, and the parametric variables of the CIEDE2000 color difference equation were equivalent to 1. The ΔE00 was assessed based on clinical perceptibility and acceptability threshold readings, which were reported in literature to be at ΔE00 ≤ 1.30 and ΔE00 ≤ 2.25 units, respectively (Ghinea et al., 2010, Taşın et al., 2020).

### 2.6 Statistical analysis

Normality was assessed through descriptive statistics, plots (Q-Q plots and histograms), and normality tests. All data demonstrated a normal distribution, leading to the adoption of parametric analysis. Two factorial ANOVA models were employed to investigate the relationship between various factors (ceramic type, thickness, staining solution, and time intervals) and color variation (ΔE_00_) as well as translucency parameter (TP_00_). Adjusted means along with 95 % confidence intervals (CIs) were computed, with a significance level set at a p-value of < 0.05. The data analysis was conducted using IBM SPSS for Windows (Version 26.0).

## 3 Results

**Fig. 2:**
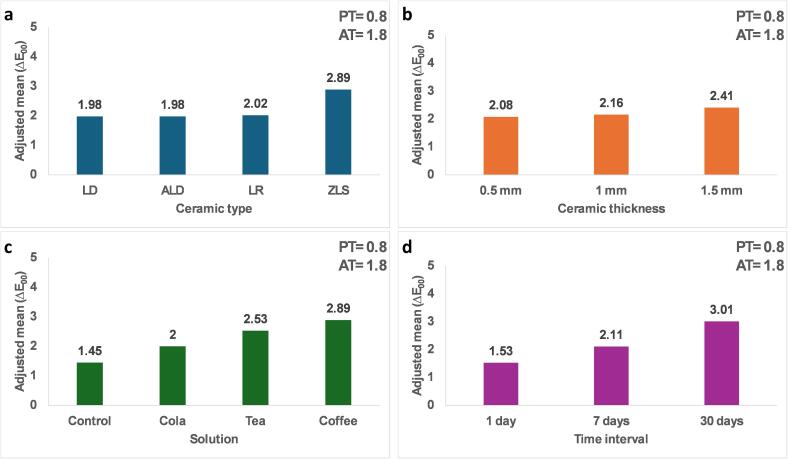
(a) Color variation (ΔE_00_) of different ceramic materials. (b) Color variation (ΔE_00_) of different ceramic thicknesses. (c) Color variation (ΔE_00_) of ceramics after immersion in different staining solutions. (d) Color variation (ΔE_00_) across time.

Table 1 and Fig. 2 represent the association of various factors with ΔE_00_ (color variation). The results revealed significant differences in ΔE_00_ values among the studied factors. In terms of dental material, ZLS exhibited significantly the highest adjusted mean ΔE_00_ (2.89), followed by LR (2.02), while LD and ALD showed the lowest ΔE_00_ values (1.98) without significant differences between them. Additionally, thickness had a significant impact, with 0.5 mm thickness (2.08) showing less color variation compared to 1 mm (2.16) and 1.5 mm (2.41). The type of staining solution also played a crucial role, with coffee leading to the highest ΔE_00_ (2.89), followed by tea (2.53), while the control (1.45) showed the least color variation. Furthermore, the time interval significantly affected color variations, with 30 days (3.01) inducing the most substantial color shift compared to 1 day (1.53) and 7 days (2.11).

**Table 1:** Factorial ANOVA for the association of different factors with ΔE_00._

		Adjusted mean (SE)	95 % CI	P value
Group	LD	1.98 (0.02) **a**	1.97, 2.06	<0.001*
	ZLS	2.89 (0.02) **b**	2.85, 2.94	
	ALD	1.98 (0.02) **a**	1.93, 2.02	
	LR	2.02 (0.02) **a**	1.93, 2.02	

Thickness	0.5 mm	2.08 (0.02) **a**	2.04, 2.12	<0.001*
	1 mm	2.16 (0.02) **b**	2.12, 2.20	
	1.5 mm	2.41 (0.02) **c**	2.38, 2.45	

Merging solution	Control	1.45 (0.02) **a**	1.40, 1.49	<0.001*
	Cola	2.00 (0.02) **b**	1.95, 2.04	
	Coffee	2.89 (0.02) **c**	2.85, 2.94	
	Tea	2.53 (0.02) **d**	2.49, 2.58	

Time	1 day	1.53 (0.02) **a**	1.49, 1.57	<0.001*
	7 days	2.11 (0.02) **b**	2.07, 2.15	
	30 days	3.01 (0.02) **c**	2.97, 3.05	

Model F: 625.61, p < 0.001*, Adjusted R^2^: 0.81.

a-d: different lowercase letters denote significant differences between groups.

**Fig. 3:**
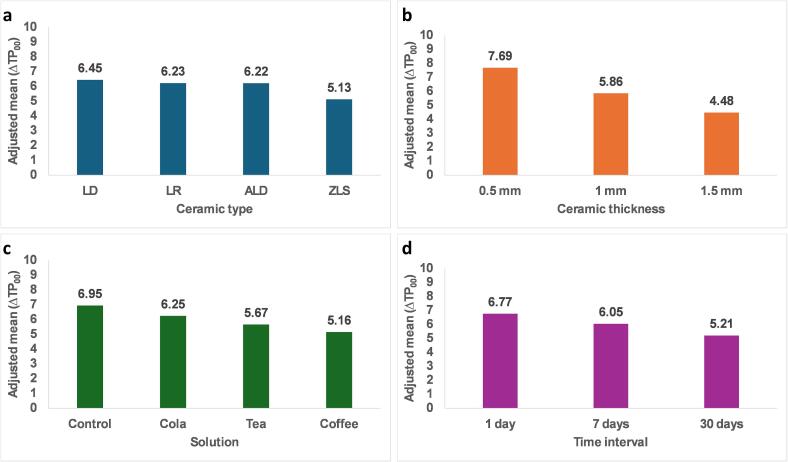
(a) Translucency parameter (TP_00_) of different ceramic materials. (b) Translucency parameter (TP_00_) of different ceramic thicknesses. (c) Translucency variation (ΔTP_00_) of ceramics after immersion in different staining solutions. (d) Translucency variation (ΔTP_00_) across time.

Table 2 and Fig. 3 represent the association of various factors with TP. The results indicate significant differences in the TP across these factors. Notably, LD demonstrated the highest adjusted mean values (6.45) followed by LR and ALD (6.23, and 6.22, respectively), while ZLS exhibited the lowest value (5.13). Only LD and LD had no significant differences in translucency unlike other tested groups. Material thickness played a crucial role, with 0.5 mm thickness showing the highest translucency (7.69), followed by 1 mm (5.86) and 1.5 mm (4.48). The type of merging solution also influenced TP, with the control group showing the highest translucency (6.95), followed by cola (6.25), tea (5.67) and coffee (5.16). Additionally, the duration of exposure, had a significant impact, with 1 day (6.77) showing the highest translucency compared to 7 days (6.05) and 30 days (5.21).

**Table 2:** Factorial ANOVA for the association of different factors with TP.

		Adjusted mean (SE)	95 % CI	P value
Group	LD	6.45 (0.03) **a**	6.40, 6.50	<0.001*
	ZLS	5.13 (0.03) **b**	5.08, 5.18	
	ALD	6.22 (0.03) **c**	6.16, 6.28	
	LR	6.23 (0.03) **c**	6.18, 6.29	

Thickness	0.5 mm	7.69 (0.06) **a**	7.57, 7.82	<0.001*
	1 mm	5.86 (0.02) **b**	5.81, 5.90	
	1.5 mm	4.48 (0.06) **c**	4.36, 4.59	

Merging solution	Control	6.95 (0.03) **a**	6.90, 7.00	<0.001*
	Cola	6.25 (0.03) **b**	6.20, 6.30	
	Coffee	5.16 (0.03) **c**	5.11, 5.21	
	Tea	5.67 (0.03) **d**	5.62, 5.72	

Time	1 day	6.77 (0.02) **a**	6.72, 6.81	<0.001*
	7 days	6.05 (0.02) **b**	6.00, 6.09	
	30 days	5.21 (0.02) **c**	5.17, 5.26	

Model F: 2118.30, p < 0.001*, Adjusted R2: 0.942.

a-d: different lowercase letters denote significant differences between groups.

## 4 Discussion

This study tested the effect of various solutions on color stability and translucency of CAD/CAM ceramic restorations with different compositions and thicknesses. The study’s null hypothesis was rejected because both the composition and thickness of ceramics affected their color and translucency after exposure to staining solutions. LD and ALD exhibited the best resistance to color variations (lowest ΔE_00_ values. In terms of translucency, LD consistently displayed the highest values. Thickness also had a significant impact, with the 0.5 mm thickness displaying minimal color variation and highest translucency. Coffee and tea caused the most pronounced staining effects with the highest ΔE_00_ values, and significantly lowest translucency values. Furthermore, longer exposure time intervals were found to contribute to reduced color stability and translucency.

Individuals vary in their ability to perceive color differences, where ΔE value ≤ 2.25 is considered clinically acceptable (Ghinea et al., 2010, Taşın et al., 2020). However, a ΔE value exceeding 3.3 is noticeable by patients and is considered undesirable clinical value (Vichi et al., 2011, Kanat-Ertürk, 2020).

The present study found that ZLS exhibited the highest ΔE_00_ value, while LD and ALD showed the lowest ΔE_00_ values. The variation between the color stability of the tested materials might be related to differences in the materials’ composition. The presence of zirconia particles in ZLS can cause greater color variations due to its porous structure that allows stain penetration (Kanat-Ertürk, 2020, Ren et al., 2012). In addition, zirconia particles could act as an abrasive agent that increase the surface roughness making the material more susceptible to discoloration (Matzinger et al., 2019, Turker and Kursoglu, 2021, Duarte et al., 2016).

The results showed similar color stability of LD, ALD and LR while LD showed the highest translucency. In agreement with the present results, previous study showed higher color stability of LD than ZLS ceramics following 2 months of immersion in coffee and tea (Kanat-Ertürk et al., 2020). Also, another study found high color stability of LD with various thicknesses (0.5, 0.7, 1, and 1.2 mm) after coffee thermocycling (Acar et al., 2016). Similarly, one study reported a variation in ZLS color at 0.5 mm thickness above the clinically acceptable threshold after thermocycling and immersion in coffee, unlike LD and monolithic zirconia (Subaşı et al., 2018). This study also found a significant effect of ceramic thickness on color stability with the least color variation observed at the smallest tested thickness of 0.5 mm (Subaşı et al., 2018). The findings of the effect of thickness on color stability were in agreement with previous studies (Acar et al., 2016, Subaşı et al., 2018).

The current results revealed variations in TP between the tested materials and at different thicknesses. In a similar study, a correlation between the material type and the translucency of multiple glass and zirconia ceramics was noticed which agrees with the current results (Wang et al., 2013). A decrease in thickness is accompanied by a higher translucency (Wang et al., 2013, Arif et al., 2019). Similarly, the present results showed the highest translucency at 0.5 mm, while the lowest translucency values were recorded at 1.5 mm thickness. In this study, the thickness of the material showed a significant impact on color variations and translucency, with 0.5 mm thickness showing the least color variation and highest translucency compared to 1 mm and 1.5 mm thicknesses. Although a previous study found that the thickness of ZLS significantly affected its color stability, LD thickness showed no effect on color stability (Subaşı et al., 2018). This difference could be attributed to variations in the methodology where the specimens used in the Subasi et al. (2018) study were glazed and exposed to coffee only as a staining solution.

In this study, the choice of staining solution also emerged as a critical factor, with coffee resulting in the highest ΔE_00_ values, followed by tea, while the control group exhibited the least color variation. These results are in a line with those of previous articles that emphasized the prominent impact of coffee on color alteration (Lauvahutanon et al., 2016, Sarıkaya et al., 2018, Liebermann et al., 2019). Coffee causes a higher color variation than tea and cola through staining by a yellow coloring pigment with different polarities (Paolone et al., 2023). Moreover, it was noted that all ΔE_00_ values increased over time, which aligns with the aforementioned results (Arocha et al., 2014, Lauvahutanon et al., 2016, Liebermann et al., 2017a, 2019b, Stawarczyk et al., 2012, Stawarczyk et al., 2016).

The findings of this study provide original information regarding the optical characterization of translucent aesthetic dental materials. One of the study limitations was that being in vitro study unlike real-life circumstances where ceramics are bonded to natural tooth structures and are subjected to various liquids. Also, there are additional factors that can impact color variations, including diet, individual preferences, and the shade of the luting cement used. To address these complexities, future studies should focus on examining the influence of other variables such as the luting cement shade, dentin color, and novel material types on the color alteration of dental CAD/CAM ceramics.

## 5 Conclusion

Material structure, thickness, staining solution type, and exposure duration significantly impacted the color stability and translucency of CAD/CAM restorations. ALD and LD exhibited the best color stability among all tested ceramic materials. LD showed the highest translucency. The ceramic thickness of 0.5 mm had the highest color stability and translucency compared to other thicknesses. Coffee exhibited the highest color variation and lowest translucency.

## CRediT authorship contribution statement

**Passent Ellakany:** Conceptualization, Investigation, Methodology, Writing – original draft, Writing – review & editing, Supervision, Validation. **Nourhan M. Aly:** Data curation, Formal analysis, Writing – review & editing. **Shahad T. Alameer:** Investigation, Methodology, Writing – original draft. **Turki Alshehri:** Investigation, Methodology, Writing – original draft. **Shaimaa M. Fouda:** Supervision, Validation, Visualization, Writing – review & editing.

## Declaration of Competing Interest

The authors declare that they have no known competing financial interests or personal relationships that could have appeared to influence the work reported in this paper.
